# Perioperative quantification of MDS-UPDRS-III tremor measurements in patients with Parkinson’s disease using accelerometry

**DOI:** 10.1007/s00702-026-03132-0

**Published:** 2026-03-11

**Authors:** Annemarie Smid, Jan Willem J. Elting, Teus van Laar, Jolien M. ten Kate, D. L. Marinus Oterdoom, Katalin Tamasi, J. Marc C. van Dijk, Gea Drost

**Affiliations:** 1 Department of Neurosurgery, University Medical Center Groningen, University of Groningen, Hanzeplein 1 HPC AB71, 9713 GZ Groningen, The Netherlands; 2 Department of Neurology, University Medical Center Groningen, University of Groningen, Groningen, the Netherlands; 3 Expertise Center Movement Disorders Groningen, University Medical Center Groningen, Groningen, The Netherlands; 4 Department of Epidemiology, University Medical Center Groningen, University of Groningen, Groningen, The Netherlands

**Keywords:** Accelerometer, Parkinson’s disease, Tremor, MDS-UPDRS, Quantification

## Abstract

Tremor in patients with Parkinson’s disease (PD) is usually evaluated using the Movement Disorder Society Unified PD Rating Scale (MDS-UPDRS). Limitations of the MDS-UPDRS include subjectivity and rater-dependency, which may be overcome by accelerometry. This study focused on relating the MDS-UPDRS hand tremor scores to an objective scoring method with 3D-accelerometry. An accelerometric algorithm to measure and classify tremor of the hands according to MDS-UPDRS criteria is proposed. Sixty-four PD patients and 64 healthy controls (matched on sex and age) were included. Two raters assessed MDS-UPDRS tremor-criteria, while accelerometry was performed at the index finger. Measurements were executed in an off-medication state, after a washout period of at least 12 h. The 3D-acceleration data included amplitude and area-under-the-curve of power in the 4–7 Hz range. Agreement between MDS-UPDRS and accelerometric scores was analyzed with Cohen’s kappa coefficient (κ). The trends between tremor amplitude and MDS-UPDRS score were consistent with the logarithmic relationship reported in previous studies. Overall, the accelerometric scores showed a substantial agreement (> 80.2%, κ ≥ 0.732) with the tremor amplitude according to MDS-UPDRS ratings. Accelerometric constancy of rest tremor (CRT) measures correlated less with MDS-UPDRS scores (R^2^ < 0.469, p < 0.001) than the other tasks. Accelerometric test–retest reliability was good to excellent (ICC ≥ 0.789, p < 0.001). MDS-UPDRS tremor tests can be reliably translated to objective accelerometric measurements. However, discrepancies were found between accelerometric CRT-measures and MDS-UPDRS ratings. Accelerometry could complement visual evaluations of tremor, aiming to reduce rater-variability of MDS-UPDRS measurements and allow for more objective monitoring of tremor in the clinical setting.

## Introduction

Tremor is a potentially disabling motor symptom of Parkinson’s disease (PD) (Bhatia et al. 2018; Tysnes and Storstein 2017; Dorsey et al. 2018; Kalia and Lang 2015; Postuma et al. 2015). It is characterized by involuntary, rhythmic and oscillatory movement (Bhatia et al. 2018). In PD, tremor is dominant in the 4–7 Hz frequency range and is generally observed unilaterally in the extremities (Wardt et al. 2020; Stouwe et al. 2016; Lee and Chan 2023). PD tremor is most prominent at rest, decreases in amplitude during voluntary movement of the affected body part, and increases during mental tasks or when voluntarily moving other body parts (Postuma et al. 2015; Wardt et al. 2020). Accurate tremor evaluations can significantly benefit PD management, considering the diverse manifestations of PD and continuously evolving treatment options.

PD tremor of the hands is most often assessed using the Movement Disorder Society Unified PD Rating Scale part III (MDS-UPDRS-III): postural tremor (3.15), kinetic tremor (3.16), rest tremor (3.17), and constancy of rest tremor (3.18) (Goetz et al. 2019). Tremor severity is rated based on the observed maximal amplitude (3.15–3.17) and presence (3.18) of tremor during the examination period (Goetz et al. 2019).

Although a substantial number of criteria is defined for the rating, not all criteria are quantitative (Evers et al. 2019; Lukšys et al. 2018; Rodríguez-Molinero et al. 2017; Post et al. 2005; Smid et al. 2023a; Kremer et al. 2023). Therefore, it might be difficult to estimate the amplitude in centimeters or to detect minor changes in tremor by eye (Smid et al. 2023a, 2022a, b; Kremer et al. 2023; Shah et al. 2017a). Consequently, these visual evaluations are prone to vary across raters (Evers et al. 2019; Lukšys et al. 2018; Rodríguez-Molinero et al. 2017; Post et al. 2005; Smid et al. 2023a; Kremer et al. 2023). Throughout the clinical caretaking process, multiple caregivers with varying levels of experience with the MDS-UPDRS-III are involved, highlighting the necessity for consistent evaluation tools (Evers et al. 2019; Lukšys et al. 2018; Rodríguez-Molinero et al. 2017).

The addition of movement sensors during tremor assessment may overcome the shortcomings of visual evaluations. Proper data analysis may result in reliable quantitative outcome measures to guide PD therapy. Unfortunately, few systems have made their way from the laboratory setting to the outpatient clinic, possibly due to their cost, size or invasiveness (Smid et al. 2022a; Espay et al. 2019; Farzanehfar et al. 2018; Hobert et al. 2014).

Tri-axial accelerometers are precise, noninvasive and relatively low-cost movement sensors that measure linear accelerations relative to the Earth’s gravitational field (Kremer et al. 2023; Smid et al. 2022a, 2023b, c, d). Their output is a 3D timeseries of accelerations expressed in gravitational units (g) in the frame of reference of the sensor (Sievänen and Kujala 2017; Karas et al. 2019). Accelerometers allow quantification of parameters that are relevant for assessing tremor (e.g., amplitude, frequency) (Yokoe et al. 2009; Smid et al. 2024). These accelerometric measures could be applied to calculate objective tremor scores based on MDS-UPDRS-III criteria (Smid et al. 2022a, b, 2023b).

Previous studies have mainly focused on quantifying tremor by merely predicting or estimating UPDRS scores using movement sensor data, without literal translation of MDS-UPDRS-III scoring criteria to accelerometric measures (Shah et al. 2017a; Rigas et al. 2016; Jeon et al. 2011; Thielgen et al. 2004; Schaeffer et al. 2018). For example, Schifino et al. used acceleration power spectral density (PSD) to detect tremors in PD and correlated these data with MDS-UPDRS-III scores (Schifino et al. 2025). Still, direct translation of the upper extremity MDS-UPDRS-III tremor items to an objective accelerometric method has not yet been described. Therefore, this study directly relates the upper extremity MDS-UPDRS-III tremor ratings to an objective method measuring tremor severity using accelerometry in a clinical setting.

## Methods

This prospective study was performed at the Parkinson Expertise Center (Punt voor Parkinson) of the University Medical Center Groningen (UMCG), the Netherlands. Exemption from the act on research involving human subjects was granted by the local medical ethics review committee (METc UMC Groningen). This study was conducted in full compliance with the Helsinki Declaration for research on human beings.

### Participants

Between July 2021 and July 2023, consecutive patients with PD in the trajectory for Deep Brain Stimulation (DBS) surgery, and age- and sex-matched healthy controls (HC) were included. Screening of the participants was performed by the primary researcher (A.S.). All participants received oral information and a participant information letter. Written informed consent was obtained from each participant. All PD patients were diagnosed according to UK Brain Bank criteria (Hughes et al. 1992) and were Hoehn and Yahr stage I–V (Hoehn and Yahr 1967). None of the HC had a current diagnosis of PD or any neurological disease. Exclusion criteria were musculoskeletal system disorders or physical disabilities, recent alcohol/drugs abuse, and psychosis or current depression. None of the participants were treated with tremor-inducing drugs during the study.

### Materials

**Fig. 1:**
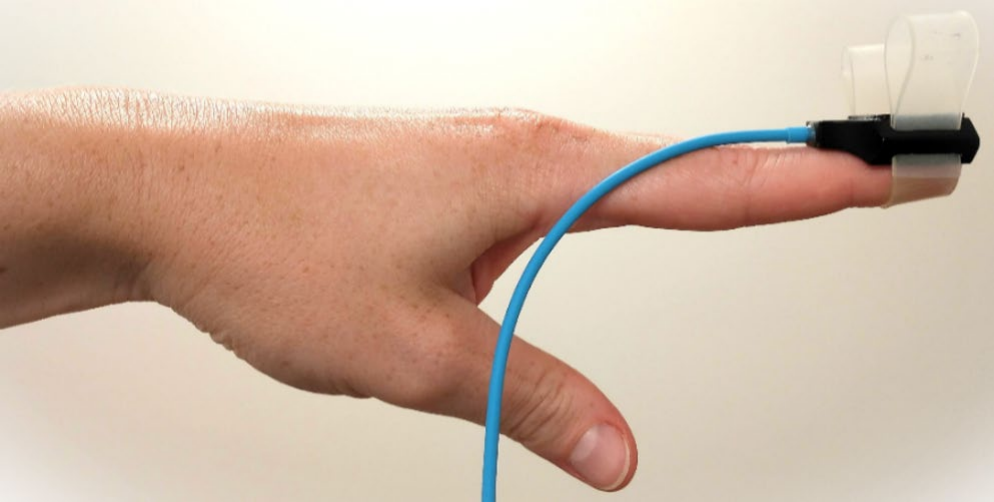
Accelerometer positioned at the tip of the left index finger

Two wired tri-axial accelerometers (MMA8452Q tri-Axis, Freescale Semiconductor, Inc., Austin, TX, USA) with a range of ± 2 g and a sampling rate of 200 Hz were used (Smid et al. 2022a, 2023b, c). The accelerometers were secured in in-house developed, non-conductive plastic cases (Smid et al. 2022a, 2023b, c). The sensors were attached to the index fingers with adjustable silicon straps (see Fig. 1). The tip of the index finger was chosen as position for the accelerometer to ensure the capture of maximal tremor amplitude (Journee et al. 2007; Jankovic 2008).

Accelerometry data were recorded with LabVIEW v. 2017 (National Instruments, Austin, TX, USA), as described previously by Smid et al. (Smid et al. 2022a, 2023b, c). Signal analysis was performed in MATLAB v. 2023a (MathWorks, Natick, MA, USA) and statistical analysis was performed in IBM SPSS statistics v. 28 (International Business Machines Corporation, New York, NY, USA). The assessment of the participants included MDS-UPDRS-III items 3.15–3.18 (Goetz et al. 2019). MDS-UPDRS part II was used to evaluate the motor aspects of experiences of daily living in the PD group (Goetz et al. 2019).

### Measurements

Healthy controls (HC) were included to determine the natural variation of the 4–7 Hz frequency range of postural tremor (PT), kinetic tremor (KT), rest tremor (RT) and constancy of rest tremor (CRT) tests. Also, HC data were used to calculate thresholds from the accelerometric data that were not quantitatively described in MDS-UPDRS-III 3.15–3.18. All measurements were conducted in an off-medication state, with a washout period of at least 12 h. If applicable, DBS devices were turned off during the measurements (at least 12 h) before testing. The accelerometers were placed at the distal phalanx of the index finger of each hand during all measurements. All accelerometry measurements were performed with the participant in a sitting position. Tests lasted ten seconds each. MDS-UPDRS-III tasks 3.15–3.17 (Goetz et al. 2019) were performed at both hands separately to assess PT, KT, RT, and CRT, respectively, while accelerometric data were recorded simultaneously. All tests were performed twice for each task, in order to determine the accelerometric test–retest reliability. Tremor severity was assessed by two MDS-UPDRS-III raters who were blinded to the accelerometric data.

### Data pre-processing

Pre-processing was performed in part as described previously (Smid et al. 2022a, 2023b, c). Calibrated raw acceleration data (g) were converted to cm/s^2^. The norm of the acceleration vector in all three directions was calculated from the root mean square of the resultant signal of the three axes (Maldonado-Naranjo et al. 2019; Salarian et al. 2007). Non-causal second-order high-pass (0.5 Hz) and low-pass (20 Hz) Butterworth filters were used to remove digital noise and higher-order harmonics (Smid et al. 2022a; Maldonado-Naranjo et al. 2019; Salarian et al. 2007), resulting in the filtered acceleration norm ($\overrightarrow{a}$).

Velocity (cm/s) was calculated from $\overrightarrow{a}$ by computing the approximate cumulative numerical integral of $\overrightarrow{a}$ over a ten-second period (2000 samples) using the trapezoidal method. In order to correct for the constant that is added to the vector due to integration, this vector was centered to compute the corrected velocity ($\overrightarrow{v}$).

Displacement (cm) was calculated from $\overrightarrow{v}$ via the same numerical integration method as described above. This vector was filtered using a non-causal second-order high-pass 1.2 Hz Butterworth filter to suppress low-frequency trends caused by integration, producing displacement vector $\overrightarrow{s}$. For KT, this high-pass filter applied a cut-off frequency of 3 Hz to suppress the amplitude of low frequent arm movements (Smid et al. 2022a; Salarian et al. 2007).

### Accelerometric outcome measures

**Fig. 2:**
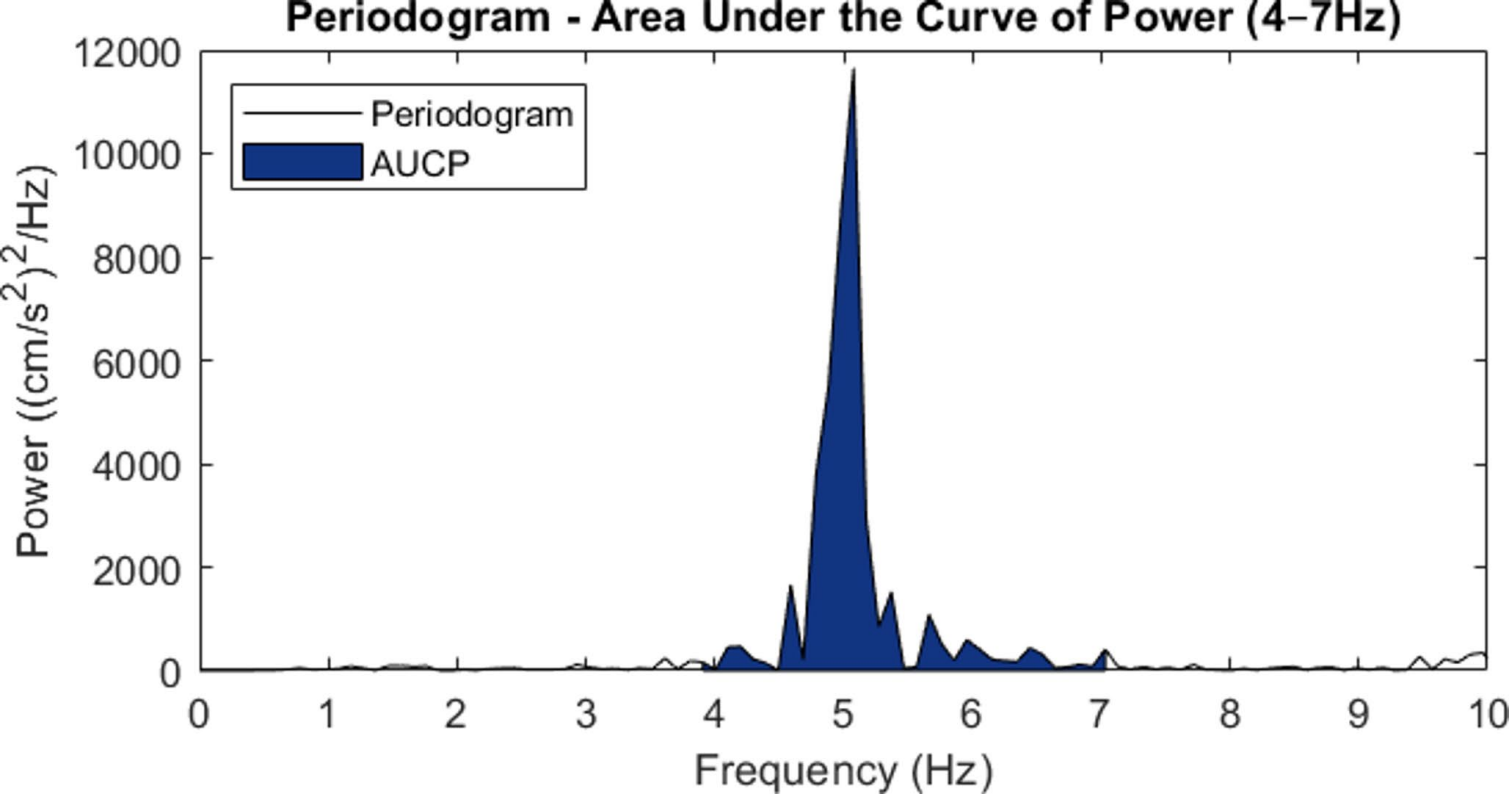
Example PSD periodogram for determination of AUCP (4–7 Hz), given in blue

**Fig. 3:**
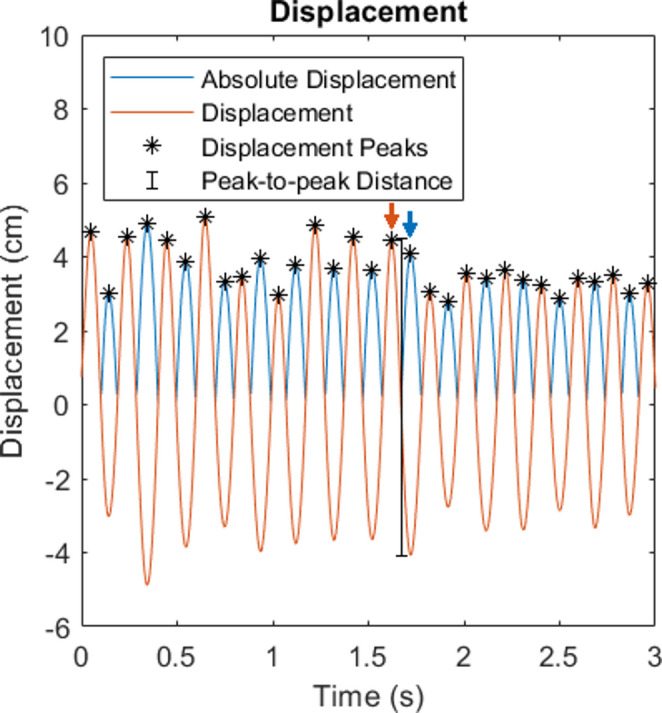
Schematic determination of maximal amplitude based on all displacement peaks of the test. A red and blue arrow mark two displacement peaks. Peak-to-peak distance is given in black lines

Two main outcome measures were calculated to assess RT, PT, and KT: (1) the area under the curve of power within the 4–7 Hz (Smid et al. 2023b) tremor frequency band (AUCP) and (2) the maximal amplitude (s_max_). The AUCP ((cm/s^2^)^2^) was calculated from the periodogram PSD estimate via trapezoidal numerical integration (Fig. 2). The s_max_ (peak-to-peak distance) of each performed task was calculated by determining all peaks in the absolute displacement ($\overrightarrow{s}$_abs_ (cm)), given in black stars (*) in Fig. 3. The mean of all computed $\overrightarrow{s}$_abs_ peaks was multiplied by factor 2 to produce s_max_.

To assess CRT, the AUCP was calculated for each second of RT, resulting in ten AUCP/s values per test. The Percentage of Time that Tremor was Present (PTTP) was calculated from the number of seconds that AUCP was above the CRT-threshold calculated from the healthy group (see Sect. "Accelerometric scores").

### Accelerometric scores

The calculated accelerometric outcome measures (AUCP, s_max_, and PTTP) are used as input for the scoring algorithm. The output of the algorithm are accelerometric (ACC) scores ranging from 0–4 per task, as MDS-UPDRS-III prescribes. The quantitative thresholds (in cm and %) provided by the MDS-UPDRS-III were used as cut-off values in the algorithm (Table 1). Other necessary thresholds were derived from accelerometric data of the HC (Table 2), according to the empirical rule (Smid et al. 2022a, 2023b, 2025). This rule states that, for normally distributed data, about 95% of the data within the population is within the population mean (*μ *) plus two times the population standard deviation (*σ *). So, the threshold (*t*) is calculated according to:

1 $$t=\mu +2\sigma $$

**Table 1:** Description of MDS-UPDRS-III 3.15–3.18 scoring criteria (Goetz et al. 2019)

Score	Severity	MDS-UPDRS-III 3.15–3.17	MDS-UPDRS-III 3.18
0	Normal	No tremor	No tremor
1	Slight	Tremor is present but less than 1 cm in amplitude	Tremor at rest is present ≤ 25% of the entire examination period
2	Mild	Tremor is at least 1 but less than 3 cm in amplitude	Tremor at rest is present 26–50% of the entire examination period
3	Moderate	Tremor is at least 3 but less than 10 cm in amplitude	Tremor at rest is present 51–75% of the entire examination period
4	Severe	Tremor is at least 10 cm in amplitude	Tremor at rest is present > 75% of the entire examination period

**Table 2:** Overview of thresholds based on HC data used for the accelerometric algorithm

Threshold	Task	Explanation	Value
PT-threshold	PT	Minimal value of AUCP for a PT test to be scored ≥ 1	396* (cm/s^2^)^2^
KT-threshold	KT	Minimal value of AUCP for a KT test to be scored ≥ 1	4441* (cm/s^2^)^2^
RT-threshold	RT, CRT	Minimal value of AUCP for an RT or CRT test to be scored ≥ 1	152* (cm/s^2^)^2^
CRT-threshold	CRT	Minimal value of AUCP per second for that second to be labelled as “tremor present”	70* (cm/s^2^)^2^

*See Sect. "Accelerometric outcome measures"

This rule was used to determine the asymptomatic thresholds for the AUCP, and thresholds for AUCP per second to determine PTTP (Table 2). The distribution of the data was determined using Kolmogorov–Smirnov test of normality (one sample K-S test).

The first step of the algorithm for all tasks is to determine whether tremor is present (Smid et al. 2022a, 2023b). Since the MDS-UPDRS-III solely states the criterium for score 0 is “no tremor”, the AUCP of each test was calculated from the accelerometric data of the HC group to determine the natural variation of noise between 4 and 7 Hz. Since healthy persons can exhibit activity in the 4–7 Hz frequency range (e.g. due to physiological tremor), AUCP-thresholding can aid in preventing physiological movements being rated as Parkinsonian tremor. An AUCP-threshold was calculated for each task (PT, KT, RT) using HC data and the empirical rule (Table 2). In the PD group, an ACC-score of 0 (“no tremor”) was given by the algorithm if the AUCP was below the calculated PT-, KT-, or RT-threshold (resp.). Otherwise, a score of 1 or higher was given, based on the amplitude (s_max_) that was measured during that test. No thresholds for s_max_ were calculated based on HC data, since these quantitative thresholds were provided in MDS-UPDRS-III.

For item 3.18, a CRT-score of 0 (“no tremor”) was given if the AUCP of the test was below the RT-threshold. Otherwise, the ACC-score for CRT was based on the calculated PTTP. The CRT-threshold was calculated from HC data based on the ten values of AUCP per second. In the PD group, a tremor was detected if AUCP per second was above the CRT-threshold. Each second that tremor was present was summed up and used to calculate the PTTP (%). The quantitative thresholds described in MDS-UPDRS-III were applied in %.

**Fig. 4:**
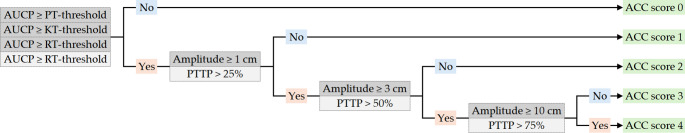
Flowchart for accelerometric score calculation, based on thresholds calculated from healthy controls and standardized MDS-UPDRS-III criteria. The pathway for PT, KT and RT are given in dark grey, while CRT is given in light grey. “No” pathways are given in blue. “Yes” pathways are given in orange

As a result, an MDS-UPDRS-based analysis of tremor, including an ACC-score (0–4) for each task, is provided (Fig. 4). All outcome measures were calculated for both the right and the left hand sensor for all corresponding tests.

### Statistical analysis

#### Primary endpoint

For each test performed, the MDS-UPDRS-III scores provided by two raters were averaged. The level of agreement between the mean MDS-UPDRS-III score and accelerometric score of each test was evaluated by computing concordance and Cohen’s weighted kappa coefficient (κ) (McHugh 2012; Ranganathan et al. 2017). As co-primary endpoint, the mean absolute error (MAE) and the root mean square error (RMSE) were calculated to determine the level of deviation of accelerometric scores from the MDS-UPDRS-III scores.

#### Secondary endpoints

As first secondary endpoint, the inter-rater reliability between the two MDS-UPDRS-III raters was analyzed using Cohen’s weighted kappa coefficient (κ).

Second, the association between accelerometric outcome measures and mean MDS-UPDRS-III scores was studied with linear regression. The mean MDS-UPDRS-III scores were used as the predictor. Contrast coding of MDS-UPDRS-III scores was defined to be orthogonal polynomial, of which the first contrast coefficient is linear trend. Consequently, analyses were performed on whether there was a linear relationship between MDS-UPDRS-III scores and the natural logarithm (ln) of the accelerometry outcome measures, as previously described (Smid et al. 2022a, 2023b; Jeon et al. 2017; Elble et al. 2006).

Third, the correlation between accelerometric outcome measures (AUCP, s_max_, PTTP) and mean MDS-UPDRS-III scores was evaluated using Spearman’s correlation coefficient (*ρ*).

Last, the test–retest reliability of the accelerometric approach was assessed based on the ICC (two-way mixed effects model, single measurement, absolute agreement) of the AUCP, s_max_, PTTP and accelerometric scores for the repeated tasks (Smid et al. 2023b, 2025; Koo and Li 2016).

## Results

Sixty-four PD patients were included in this study (32 men, 32 women; age (mean ± SD): 61.8 ± 8.6 years) versus 64 HCs (32 men, 32 women; age (mean ± SD): 60.9 ± 8.9 years). Median motor impairment of PD patients was an MDS-UPDRS II rating of 15 (range: 3–36) out of 52. The Hoehn and Yahr stage (mean ± SD) in the PD group was 2.6 ± 0.9, and the average disease duration (mean ± SD) was 9.1 ± 4.8 years. In 23 patients, a DBS device was present. At the time of the assessment, these participants had been treated for 10.1 ± 4.3 months on average (± SD). In all PD patients and HC, 256 PT-, 256 KT-, and 256 RT-tests were performed (twice left-handed, and twice right-handed).

### Clinical MDS-UPDRS-III ratings

Cohen’s κ coefficient showed moderate to substantial inter-rater reliability of the two MDS-UPDRS-III raters for all tests (κ ≥ 0.444) (McHugh 2012). κ was 0.560 (95% CI 0.468, 0.652; p < 0.001) for PT. κ was 0.444 (95% CI0.350, 0.538; p < 0.001 for KT. κ was 0.558 (95% CI 0.478, 0.638; p < 0.001) for RT. κ was 0.489 (95% CI 0.379, 0.599; p < 0.001) for CRT. Lastly, κ was 0.512 (95% CI 0.435, 0.589; p < 0.001) for all tasks combined.

### Accelerometric outcome measures

**Fig. 5:**
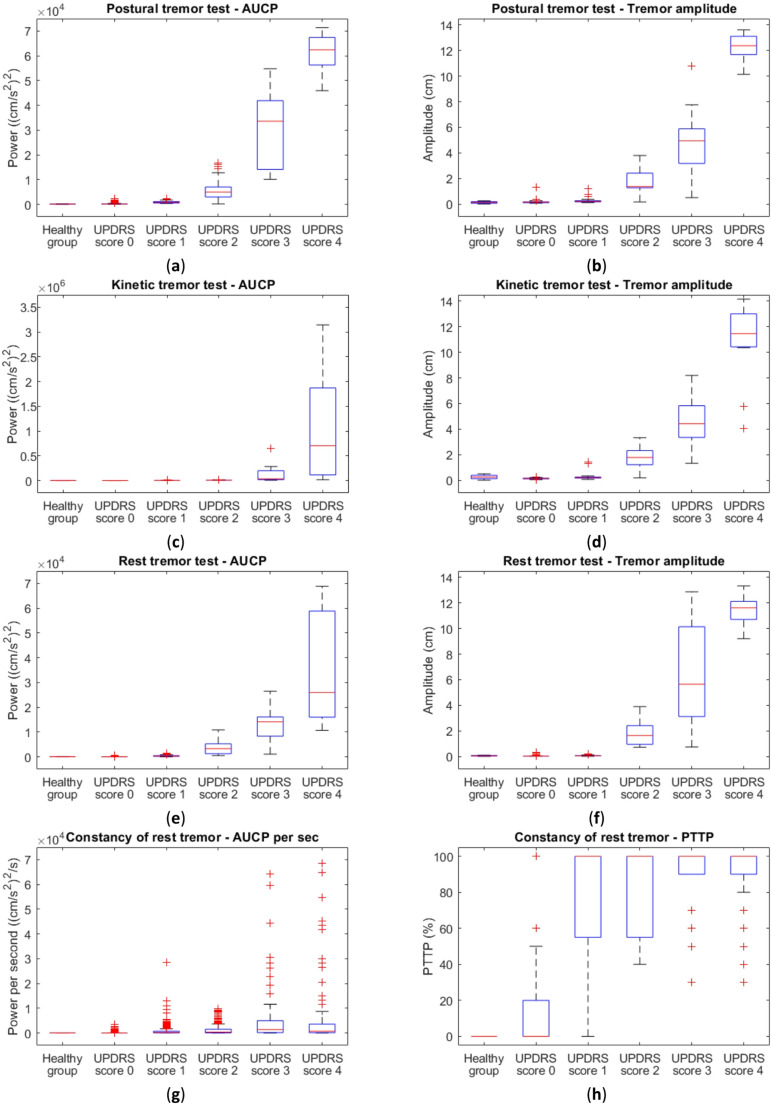
Boxplots of the AUCP of PT (**a**), KT (**c**), and RT (**e**), and of the s_max_ of PT (**b**), KT (**d**), and RT (**f**), and of the AUCP per second of CRT (**g**), and of the PTTP of CRT (**h**) in the HC and the patient group, per MDS-UPDRS-III score. Outliers are marked with red crosses ( +)

The main accelerometric outcomes were AUCP, s_max_, and PTTP, which were plotted against the averaged MDS-UPDRS-III ratings per task in Fig. 5. With increasing MDS-UPDRS-III scores, an increase in AUCP and tremor amplitude can be observed for all tremor amplitude tasks. AUCP per second and PTTP also increased with increasing MDS-UPDRS-III scores for CRT. Figure 5.g shows a prominent number of outliers for AUCP per second.

All accelerometric outcomes were natural log (ln) transformed for regression analysis, except for PTTP, as this outcome measure contained values of zero. The ln-transformed measures and PTTP were regressed on the mean MDS-UPDRS-III scores. There was strong evidence against the null hypothesis that the log-transformed outcome measures were not linearly related to the MDS-UPDRS-III scores (p < 0.001) for all tasks, see Table 3. AUCP and AUCP per second tended to increase as an ln-function of MDS-UPDRS-III (R^2^ > 0.518).

**Table 3:** Regression analysis results

Task	Measure	R	R^2^ *	Coefficient**	95% CI	*p*
PT	ln(AUCP)	0.833	0.693	9.343	8.571, 10.114	< 0.001
	ln(s_max_)	0.778	0.606	3.140	2.826, 3.454	< 0.001
KT	ln(AUCP)	0.773	0.598	5.376	4.829, 5.923	< 0.001
	ln(s_max_)	0.739	0.546	2.213	1.962, 2.463	< 0.001
RT	ln(AUCP)	0.871	0.759	11.855	11.025, 12.685	< 0.001
	ln(s_max_)	0.839	0.705	5.443	5.006, 5.880	< 0.001
CRT	ln(AUCP/s)	0.720	0.518	4.228	3.473, 4.984	< 0.001
	PTTP	0.766	0.587	5.733	5.137, 6.330	< 0.001

*Coefficient of determination; **Contrast coefficient testing for linear trend

Table 4 shows the correlations between accelerometric outcome measures (AUCP, s_max_, PTTP) and MDS-UPDRS-III scores. A good correlation was found for s_max_ in all tasks (p < 0.001). All correlations between AUCP and MDS-UPDRS-III scores were good (p < 0.001). The remaining measures showed moderate correlation with MDS-UPDRS-III scores (p < 0.001) (Koo and Li 2016).

**Table 4:** Spearman’s correlation between accelerometric outcome measures and MDS-UPDRS-III scores in the patient population

Task	Measure	Spearman’s *ρ*	R^2^	95% CI	*p*
PT	AUCP	0.810	0.656	0.762, 0.850	< 0.001
	s_max_	0.786	0.618	0.713, 0.848	< 0.001
KT	AUCP	0.830	0.689	0.765, 0.884	< 0.001
	s_max_	0.759	0.576	0.681, 0.825	< 0.001
RT	AUCP	0.845	0.714	0.805, 0.878	< 0.001
	s_max_	0.802	0.643	0.751, 0.843	< 0.001
CRT	AUCP/s	0.685	0.469	0.611, 0.747	< 0.001
	PTTP	0.715	0.511	0.647, 0.772	< 0.001

### Accelerometric scores

**Fig. 6:**
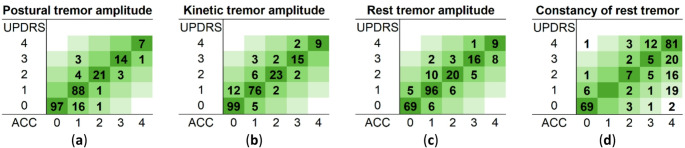
Contingency tables of the mean MDS-UPDRS-III (UPDRS) scores and calculated accelerometry (ACC) scores of the PT (**a**), KT (**b**), RT (**c**) and CRT (**d**) in the patient group. The clinical UPDRS scores are given on the y-axis, and the ACC-scores calculated by the algorithm are given on the x-axis. Matching scores are given in the dark green diagonal, neighboring scores are given in light green, and discrepant pairs are given in the lightest shades

All HC data required for threshold calculation were normally distributed according to K–S test results (p < 0.001). Table 2 shows the values of thresholds calculated from HC data. The contingency tables of mean MDS-UPDRS-III scores and ACC-scores in the patient population are given in heatmaps in Fig. 6 for all tasks.

Table 5 shows concordance and Cohen’s κ coefficient of agreement between the mean MDS-UPDRS-III ratings and the accelerometric scores per task, as well as the mean error (MAE and RMSE). Concordance between the MDS-UPDRS-III ratings and the ACC-scores was substantial for all tasks (≥ 63.3%). Cohen’s κ results showed a moderate to substantial agreement for all tasks (McHugh 2012; Ranganathan et al. 2017; Koo and Li 2016). The MAE (range 0.129–0.188) indicates that the ACC-scores differed less than a quarter point from the mean MDS-UPDRS-III ratings on average for PT, KT and RT.

**Table 5:** Agreement between MDS-UPDRS-III and ACC-scores for MDS-UPDRS-III items 3.15–3.18

Task	Concordance	Cohen’s κ	95% CI	*p*	RMSE (95%CI)	MAE (95%CI)
3.15 PT	88.7%	0.830	0.773, 0.887	< 0.001	0.400 (0.353, 0.447)	0.129 (0.108, 0.150)
3.16 KT	86.7%	0.805	0.744, 0.866	< 0.001	0.410 (0.363, 0.457)	0.145 (0.121, 0.169)
3.17 RT	82.0%	0.744	0.679, 0.809	< 0.001	0.451 (0.401, 0.502)	0.188 (0.152, 0.214)
3.18 CRT	63.3%	0.468	0.394, 0.542	< 0.001	1.204 (1.078, 1.330)	0.652 (0.596, 0.708)
All tasks	80.2%	0.732	0.701, 0.763	< 0.001	0.704 (0.673, 0.735)	0.278 (0.262, 0.294)

### Accelerometric reliability

In Table 6, the test–retest reliability of both the MDS-UPDRS and the accelerometric method is given for the accelerometric outcome measures and the calculated ACC-scores of the repeated tasks. Accelerometric reliability was excellent for both the s_max_ and ACC-score of RT, and the remaining ICCs showed good test–retest reliability. The clinical approach (MDS-UPDRS-III score) showed moderate to good test–retest reliability (ICC) (Koo and Li 2016).

**Table 6:** Test–retest reliability (ICC: two-way mixed effects model, single measurement, absolute agreement)

MDS-UPDRS-III task	Outcome measure	ICC	95% CI	*p*
3.15 Postural tremor of the hands	AUCP	0.800	0.729, 0.871	< 0.001
	s_max_	0.815	0.745, 0.867	< 0.001
	ACC-score	0.815	0.744, 0.867	< 0.001
	MDS-UPDRS score	0.696	0.578, 0.814	< 0.001
3.16 Kinetic tremor of the hands	AUCP	0.789	0.694, 0.879	< 0.001
	s_max_	0.889	0.844, 0.921	< 0.001
	ACC-score	0.866	0.813, 0.904	< 0.001
	MDS-UPDRS score	0.766	0.719, 0.813	< 0.001
3.17 Rest tremor amplitude of the hands	AUCP	0.870	0.819, 0.907	< 0.001
	s_max_	0.928	0.898, 0.949	< 0.001
	ACC-score	0.920	0.887, 0.943	< 0.001
	MDS-UPDRS score	0.829	0.741, 0.917	< 0.001
3.18 Constancy of rest tremor of the hands	AUCP/s	0.869	0.818, 0.907	< 0.001
	PTTP	0.868	0.811, 0.907	< 0.001
	ACC-score	0.869	0.817, 0.907	< 0.001
	MDS-UPDRS score	0.664	0.593, 0.735	< 0.001
Tasks 3.15–3.17 combined	AUCP	0.891	0.849, 0.927	< 0.001
	s_max_	0.909	0.888, 0.927	< 0.001
	ACC-score	0.917	0.901, 0.931	< 0.001
	MDS-UPDRS score	0.831	0.778, 0.884	< 0.001
All tasks combined	ACC-score	0.899	0.879, 0.916	< 0.001
	MDS-UPDRS score	0.736	0.687, 0.785	< 0.001

## Discussion

This study describes the relation of MDS-UPDRS-III hand tremor assessments to an objective accelerometric method based on the MDS-UPDRS-III using thresholds calculated from healthy participant data. This study proposes a quantitative scoring algorithm for MDS-UPDRS-III tests 3.15–3.18 using accelerometry. The agreement between clinically scored MDS-UPDRS-III ratings and objective scores calculated from accelerometric data was studied, showing that MDS-UPDRS-III hand tremor tests can be accurately quantified with accelerometry, although the algorithm still needs to be optimized in order to provide better agreement. Trends were consistent with the logarithmic relationship between tremor amplitude and MDS-UPDRS-III scores described in previous studies, for both amplitude and AUCP as measured by the accelerometer (Kremer et al. 2023; Smid et al. 2022a, 2023b; Elble et al. 2006, 2017; Wastensson et al. 2013). However, this study also showed discrepancies between accelerometric CRT-measures and clinically scored MDS-UPDRS-III ratings, particularly in the agreement between ACC- and clinical scores.

The primary contribution of this study is relating MDS-UPDRS-III hand tremor measurements to an objective scoring algorithm using accelerometry in a clinical setting, using the gold standard as a basis. In addition, HC were included to determine the natural variation of the accelerometric outcome measures and to provide the thresholds needed for the algorithm that were not stated in the MDS-UPDRS-III. The HC group was matched for age and sex with the PD group, and two certified MDS-UPDRS raters assessed all participants in order to correct for the effects of inter-rater variability. The ACC-scores generated by the algorithm are straightforward to understand, as they are based on the same MDS-UPDRS-III criteria that PD caregivers are already familiar with.

The main limitation of this study is that the gold standard used in this study is flawed to a certain extent. Even though the MDS-UPDRS-III is the most standardized and most used method for measuring upper extremity Parkinsonian tremor, amplitudes are difficult to assess visually. An imperfect gold standard limits the maximum achievable agreement of novel methods with the gold standard. Two experienced raters were employed for this study, and still inter-rater reliability was not perfect (κ ~ 0.44–0.56), which is in line with previous studies (Kremer et al. 2023; Shah et al. 2017a; Smid et al. 2022a; Yokoe et al. 2009). This implies that rating the same patient at the same moment in the same condition will result in different assessments per rater, which complicates using the MDS-UPDRS-III as the gold standard.

### Clinical MDS-UPDRS-III ratings

This study shows a moderate to substantial inter-rater reliability of the MDS-UPDRS-III ratings. Comparable values have been observed in other studies, highlighting the importance of a more uniform approach to perform MDS-UPDRS-III assessments (Evers et al. 2019; Post et al. 2005; Smid et al. 2022a, 2024). Future statistical analyses should consider mixed models to handle clustering by subject, hand, and rater.

### Accelerometric outcome measures

Figure 5 and Table 3 illustrate that the trends between tremor amplitude and MDS-UPDRS-III scores were consistent with the logarithmic relationship reported in previous studies (Kremer et al. 2023; Smid et al. 2022a, 2023b; Elble et al. 2006, 2017; Wastensson et al. 2013). Overall, good correlations were found between accelerometric outcome measures and MDS-UPDRS-III scores, indicating that the algorithm comes to similar conclusions as the MDS-UPDRS-III raters. However, the accelerometric constancy of rest tremor (CRT) measures showed only moderate correlation with MDS-UPDRS scores (R^2^ < 0.469, p < 0.001), likely due to the calculation of CRT in the 10-s time window when RT was performed, instead of continuously during the whole examination. The choice to only use accelerometric data that was certainly performed in the RT position to calculate CRT was made to ensure correct calculation of CRT-scores based on accelerometric data captured in a controlled setting. However, this might have led to a discrepancy between the input for the MDS-UPDRS-III scores and the input for the accelerometer. To improve the real-life application of the accelerometric algorithm, its agreement with MDS-UPDRS-III results should be compared to ACC-results based on continuous data in RT position as input for the algorithm. An explanation for the outliers shown in Fig. 5.g is the grouping of continuous accelerometric data (AUCP per second) according to ordinal MDS-UPDRS-III, where all ten values per test are grouped under a single rating. For example, MDS-UPDRS-III CRT-score 1 indicates that tremor is absent 51–75% of the time, meaning that at least 25% of accelerometric values of that test are above the threshold, probably resulting in outliers. With increasing tremor severity and constancy, AUCP per second values tends to increase as well. Although investigating CRT over a longer period of time might not directly solve this issue, it will allow a more granular analysis of the time that tremor was present.

### Accelerometric scores

Table 5 and Fig. 6 show substantial agreement between MDS-UPDRS-III ratings and ACC-scores for PT, KT, and RT (McHugh 2012; Ranganathan et al. 2017). The lower agreement between MDS-UPDRS-III and ACC-scores for CRT can be explained by the provided MDS-UPDRS-III scores being mainly dichotomous, as there was a high density of CRT-scores 0 and 4, inherent to CRT-ratings. The MDS-UPDRS-III CRT-score automatically defaults to 0 when no rest tremor is observed, either by the raters or the algorithm. In this dataset, there were several instances where the mean MDS-UPDRS-III score was 1–3 and the ACC-score was 4 for CRT. This could be explained by the accelerometer detecting minor tremors not visible for the naked eye. Previous studies have noted that suboptimal correlations between accelerometry and clinical evaluations can arise from subtle movement changes being challenging to estimate visually (Smid et al. 2022a, 2023b; Yokoe et al. 2009; Shah et al. 2017b).

Also, the threshold of 70 (cm/s^2^)^2^ for determining CRT might be suboptimal, despite being based on data of healthy subjects. Considering the ceiling effect that is displayed in Fig. 6.d, it would be reasonable to investigate higher CRT-thresholds in future research.

Figure 6a shows an overestimation of MDS-UPDRS-III PT score 0 being rated as score 1 by the accelerometer. This phenomenon is less strong for KT. This might be explained by KT-data undergoing 3 Hz high-pass filtering to suppress the amplitude of low frequent arm movements, while this cut-off was set at 1.2 Hz for PT. High-pass filtering with e.g. 2 Hz cut-off might suppress movements caused by muscle contraction while holding the arms in position during the PT task.

### Accelerometric reliability

The test–retest reliability of the accelerometric method was good to excellent for all outcome measures and ACC-scores, underlining its potential to allow objective monitoring of hand tremor. Comparable levels of reliability have been reported in previous studies (Smid et al. 2023b, 2025). Overall, it stood out that MDS-UPDRS-III test–retest reliability was consistently lower than accelerometric reliability for all tasks.

### Future perspectives

The field of quantifying PD motor skills using movement sensors is evolving (Evers et al. 2025; Keba et al. 2025). The notion that inter- and intra-rater variability of the MDS-UPDRS should be reduced, and assessments should be performed uniformly, is shared among PD experts worldwide (Espay et al. 2019; Elble and Ondo 2022; Mahadevan et al. 2020). Minimizing variability in tremor assessment will enhance the accuracy of evaluating disease progression and treatment effects. The clinical context of the proposed technique is quantification of tremor assessments in PD patients undergoing stereotactic neurosurgery, although such a method could also be expedient during standard follow-up. Future research should focus on the implementation of a real-time algorithm during motor assessments at the outpatient clinic. The algorithm proposed in this paper could also address a clinical challenge faced by physicians when visually scoring other tremor syndromes (e.g. Essential tremor). With relatively minor adjustments, the frequency range and parameters used in the current scoring algorithm could be tailored to align with standardized rating scale criteria for other tremor types, e.g. the Fahn-Tolosa-Marin Clinical Rating Scale for Tremor and The ET Rating Assessment Scale (Elble et al. 2012; Fahn et al. 1988).

To support clinicians in improving patient outcomes, it is essential to present these complex quantitative data in a format that is easy to interpret. Addressing big data problems will become increasingly manageable with the rise of machine learning and artificial intelligence. Examples include neural networks and support vector machines, which allow effective management of large volumes of multidimensional data (Jeon et al. 2017; Evers et al. 2025; Espay et al. 2016; Heldman et al. 2014; Liu et al. 2022; Channa et al. 2022). However, the internal workings of these tools might not be transparent due to black box algorithms. Therefore, the method proposed in this manuscript is a form of non-black box programming, where each analysis step and threshold to produce the output is known. This way, there is transparency on how the algorithm arrives at its outcome measures, and the output can be validated. Future research should focus on optimizing the accelerometric thresholds of the proposed algorithm. It is recommended to validate the proposed algorithm with a second cohort against multiple levels of PD severity, and adjust thresholds according to possible tremor variability observed in early-stage PD. Moreover, the influence of medication (e.g. medication-induced dyskinesias) on accelerometric results should be investigated in the future. This could improve the generalizability of future results, since the included patients for this study do not represent the entire PD population. Further investigating the variability of MDS-UPDRS-III measures and accelerometric outcome measures is recommended to optimize this technique.

Future development of the method presented in this paper should also focus on quantifying the clinical evaluation of PD therapies, and continuous data collection in real-life environments that reflect the patient's daily functioning. These data could be used to generate personalized feedback for clinicians, caregivers, and patients, which will support individualized treatment approaches and enhance self-management of the patient.

## Conclusion

This study showed that the MDS-UPDRS-III tremor tests can be quantified using accelerometry with high reliability, providing clinically meaningful outcomes. The accelerometric data were consistent with the logarithmic relationship reported in previous studies. Agreement between the clinically scored MDS-UPDRS-III ratings and accelerometric scores was substantial. Discrepancies in the constancy of rest tremor were found, for which recommendations were formulated. The proposed method has the potential to reduce rater dependency of MDS-UPDRS-III measurements and to allow for more uniform assessment of hand tremor in PD.

## Data Availability

No datasets were generated or analysed during the current study.
